# Young adult cancer survivors and work: a systematic review

**DOI:** 10.1007/s11764-017-0614-3

**Published:** 2017-05-06

**Authors:** Dawn S. Stone, Patricia A. Ganz, Carol Pavlish, Wendie A. Robbins

**Affiliations:** 1 School of Nursing, University of California Los Angeles, Los Angeles, USA; 2 David Geffen School of Medicine, University of California Los Angeles, Los Angeles, USA; 3 Fielding School of Public Health, University of California Los Angeles, Los Angeles, USA

**Keywords:** Young adult cancer survivors, Work, Employment, Occupational health

## Abstract

**Context:**

Sixty-three percent of cancer survivors continue to work, or return to work after treatment. Among this population, work ability and challenges encountered in the workplace by *young* adult cancer survivors have not been well established.

**Purpose:**

The purposes of the study are to describe what is currently known about work-related issues for young adult cancer survivors diagnosed between ages 15 and 39, to identify gaps in the research literature, and to suggest interventions or improvements in work processes and occupational settings.

**Methods:**

A narrative review of articles using PubMed, CINAHL, and PsychInfo was conducted without date limitations. Search phrases included young adult cancer survivors, long-term cancer survivors, young adults affected by cancer, further combined with key terms employment, work, and occupationally active. Inclusion criteria for publications were young adult cancer survivors initially diagnosed between the ages of 15 and 39, data about work or employment was presented, and articles written in English.

**Results:**

Twenty-three publications met the inclusion criteria. Work-related issues included the potential for reduced work productivity from cancer-changed physical and cognitive functional ability that affected income, and resulted in distress. Coping style, support systems, and changing perspectives about work and life in general were also influential on career decisions among young adult cancer survivors.

**Conclusions:**

More research is needed to study interventions to better manage health changes in young adult cancer survivors within the context of the workplace. Since financial hardship has been shown to be especially high among young cancer survivors, employment is essential to ensure payment of cancer-associated costs and continued medical care.

**Implications for Cancer Survivors:**

While young adult cancer survivors may initially grapple with cancer-related physical and psychosocial changes that impact work productivity or influence choice of occupation, employment appears to enhance overall quality of life.

## Workplace health and safety considerations for young adult cancer survivors

Young adults, initially diagnosed with cancer between the ages of 15 and 39 [1], may look forward to a lifetime of opportunities. The number of people living beyond a cancer diagnosis reached nearly 15.5 million in 2014 and is expected to rise to almost 19 million by 2024 [2]. Considering this anticipated increase in cancer survivors, many will be at an age when cancer and its treatments could alter employment opportunities. With earlier cancer diagnoses among younger working-aged persons, job-related accommodations could have far-reaching social and economic effects [3]. The ability to work following cancer treatment is important for maintaining self-respect, identity, and living conditions. It is also important for society to keep people employed for economic reasons and to prevent social inequality [4]. Information about adults who continue to work long after a cancer diagnosis is integral to understanding their potential health and safety needs in the workplace [3].

A life course perspective on cancer can advance understanding of the unique ways cancer affects young adults [5]. Cancer survivors encounter a variety of work experiences such as changes in responsibilities, decreased capacity to work, and perhaps job loss. Work ability is a complex concept that changes over time as a new balance between job demands and personal capacity is established [6]. These changes can be associated with cancer or cancer treatment, but survivors may also voluntarily change employment after self-reflection about life’s priorities [7]. Based upon issues associated with developmental stages coupled with confronting a serious disease, early questions preliminary to this review pondered how young adult cancer survivors approach and interact with others within the work environment. Disclosure, career choices, impact of cancer treatments on health, and work ability over the course of a career could influence employment status. We also wondered how occupational and environmental health professionals could support working cancer survivors. Hence, this review provides a comprehensive analysis of what is known about young adult cancer survivors and employment. Young adult survivors are an understudied population compared with other age groups who undergo complicated journeys because of their life stage [8]. Therefore, the purposes of this analysis of scholarly literature are to examine what is currently known about the work-related issues for young adult cancer survivors diagnosed between ages 15 and 39, to identify gaps in the research literature, and to suggest interventions or improvements in work processes and occupational settings.

## Methods

### Search strategy

The first author searched the literature using the following electronic databases: PubMed (United States National Library of Medicine); The Cumulative Index to Nursing and Allied Health Literature (CINAHL); and PsychINFO (American Psychological Association). The search was conducted in English without date restrictions and concluded in January 2016. Search phrases included young adult cancer survivors, long-term cancer survivors, and young adults affected by cancer. Search phrases were combined with key terms employment, work, and occupationally active. Eligible publications were also hand-searched for additional references.

### Eligibility criteria

The following criteria were used to select publications for this review: (1) Inclusion of young adults initially diagnosed with cancer between the ages of 15 and 39 [1] at any time during survivorship. The age range during survivorship may vary based on how long after diagnosis the research was conducted. (2) Inclusion of data about work or employment (3) articles written in English.

### Review strategy

**Fig. 1:**
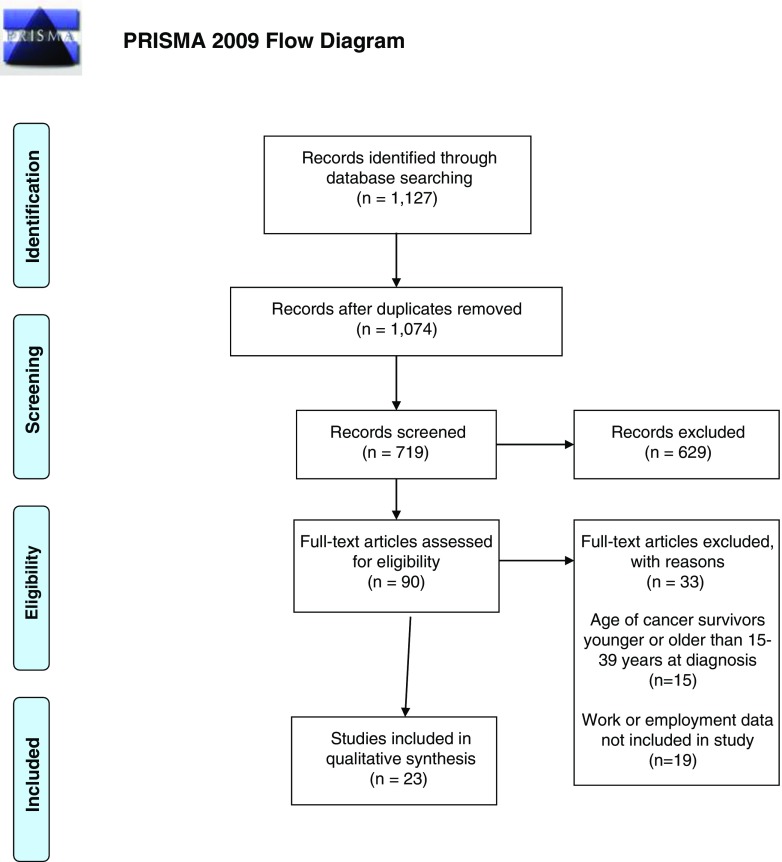
PRISMA (2009) flow diagram

One thousand one hundred twenty-seven articles were identified from PubMed, CINAHL, and PsychINFO databases using the search terms listed earlier; 53 were duplicates (Fig. 1). Study eligibility included the following: age of cancer survivors at initial diagnosis as well as length of time since diagnosis or treatment; inclusion of work or employment issues as part of content. Study exclusions: samples of adults diagnosed with cancer at a mean age of 40 or older; age at diagnosis unknown. Careful consideration was given to investigations listing age categorically to determine if the sample met the eligibility criteria. However, quite often these studies did not align the findings with the age categories. The US Department of Labor’s Fair Labor Standards Act defines workers who are economically dependent on the business of an employer, regardless of skill level, to be considered employees. Whereas, independent contractors are workers with economic independence who are in business for themselves [9]. Number of work hours in any capacity are not part of the US definitions of employment; hence, this review considered all variations in reported employed and self-employed work as determined by the research reviewed.

### Quality assessment

Quality was assessed using the *Johns Hopkins Hospital/The Johns Hopkins University Evidence Level and Quality Guide* [10]. Articles were scored according to evidence levels based upon type of article or research design. Three of the 23 (13%) publications were at level I: experimental study, randomized controlled trial (RTC), or systematic review of RCTs with or without meta-analysis [11–13]. Three articles (13%) were at level II: quasi-experimental studies, systematic review of a combination of randomized controlled trials and quasi-experimental studies, or quasi-experimental studies only, with or without meta-analysis [14–16]. The remaining 17 publications were level III: non-experimental study, systematic review of a combination of RCTS, quasi-experimental, and non-experimental studies only, with or without meta-analysis, or qualitative study or systematic review of qualitative studies with or without meta-synthesis [8, 9, 17–31]. Levels IV (opinions of respected authorities, committees, and consensus panels) and V (quality improvement program evaluation, case reports) provided interesting insight and background into young adult cancer survivors and work but were not included in this review.

Quality guides associated with evidence levels 1–3 include high quality: consistent, generalizable results; sufficient sample size for study design; adequate control; definitive conclusions; consistent recommendations. Good quality: reasonably consistent results; sufficient sample size for study design; some control; fairly definitive conclusions; reasonably consistent recommendations. Low quality: little evidence with inconsistent results, insufficient sample size for study design (Table 1).

**Table 1:** Publications meeting eligibility criteria concerning young adult cancer survivors (aged 15–39 at diagnosis) and work (paid employment)

First author, year; evidence level and quality rating^a^	Population and eligibility	Study design and purpose, aims, or objectives	Participant characteristics	Relevant measures	Outcomes related to work
Aksnes, 2007 Level II—A	Scandinavian sarcoma group *N* = 75 eligible 58 responses (77%) 31 male and 27 female Extremity bone tumor survivors Mean age at diagnosis: Males 20; females 16 Age at survey: Males 34 (9.4) Females 27 (4.8) Years since diagnosis: Males 14 (4.5); Females 11 (4.8) 5 years or more after treatment	Cross-sectional, comparative Purpose: To compare quality of life, fatigue, and mental distress in extremity bone tumor survivors with Hodgkin’s disease survivors, testicular cancer survivors, and gender and age-matched individuals from the general population	Cancer sites: Extremity bone tumors (EBT); Hodgkin’s disease (HD); testicular cancer (TC) Working status: Employed 45 Not employed 12	Fatigue questionnaire Hospital anxiety and depression scale Short form-36 to measure quality of life Short form-36 physical component summary scale and mental component summary scale	Extremity bone tumor survivors did not differ from the other survivor groups as to education and employment status. Extremity bone tumor survivors had lower scores on all physical dimensions of the short form-36 compared to other survivors and normative samples (*p* < 0.5) In multivariate analyses of the combined survivor and normative data, a low level of education and not being employed were associated with mental distress (OR 2.28; CI 1.26–4.14; *p* = 0.01). Extremity bone tumor survivors had higher mean fatigue scores than the normative samples (*p* < 0.5) but about the same levels as other survivor groups. Clinical implications: eventually prepare for reduced physical functioning
Bellizzi, 2012 Level III—B	National Cancer Institute Surveillance, Epidemiology, and End Results Program Adolescent and Young Adult Health Outcomes and Patient Experience Study *N* = 523 Mean age 29 ± 6.7 years 1/3 of AYAs in all 3 age groups: 15–20 years 33.8% 21–29 years 39.9% 30–39 years 38.2% <14 months after diagnosis	Cross-sectional Objectives: To identify the negative and positive impact of cancer on developmental aspects of adolescence and young adulthood and to examine these impacts according to stage of development (age at diagnosis)	Cancer sites: Germ cell, non-Hodgkin lymphoma, Hodgkin lymphoma, acute lymphocytic leukemia, or sarcoma	Life domains: Future Body appearance Control over life	The most prevalent negative life domains reported were specific to a plan: financial situation, plans for having children, plans for working, as well as body appearance and sense of control over life. With regard to a plan, all 3 age groups reported that cancer had a similar level (∼46% of the sample) of positive impact on plans and goal setting. These findings demonstrate the coexistence (in the aggregate) of negative and positive psychosocial aspects of cancer in adolescents/young adults.
Bieri, 2008 Level III—B	University Hospital of Geneva *N* = 124 patients in remission after allogeneic hematopoietic stem cell transplant (HSCT). Comparison with health controls. Median age 34 years (range 14–65) Median time from hematopoietic stem cell transplant was 7.3 years. Median age at time of answering questionnaire: 42 years. >5 years after diagnosis Free of disease at the time of evaluation	Cross-sectional Aim: To assess health-related quality of life in comparison with healthy controls	Cancer sites: AML ALL CML CLL Myelodysplasic syndrome Myeloma Myeloproliferative syndrome Aplastic anemia	Functional assessment of cancer therapy scale with specific modules for bone marrow transplant and the European Organization for Research and Treatment of Cancer (EORTC QLQ-C30) Questionnaires	In total, 119 (96%) had an occupation or were in school or training before hematopoietic stem cell transplant. After hematopoietic stem cell transplant, 60% of these patients returned to their activities, 29% returned to full-time employment, 21% part time, and 10% returned to training or school. In total, 40% of the patients did not return to work and depend on disability insurance (39%) or are retired (1%). Among patients fully employed, 73% reported good quality of life as opposed to 22% of those on disability insurance and 28% of those on part-time work (*p* < 0.0001).
Davidoff, 2015 Level I—A	US national household survey *N* = 2527 cancer survivors age 18–64 years 24.4% of the sample was in the young adult age category 18–44 Variable length of time since treatment	Descriptive Baseline prior to Affordable Care Act (ACA) for future comparison post implementation ACA Purpose: To characterize coverage options for nonelderly adult cancer survivors and the subset with financial hardship	Cancer sites: Breast Prostate Colorectal Other male genitourinary Hematologic malignancy Bone, muscle, soft tissue	Medical Expenditure Panel Survey Household Component (MEPS-HC) data from 2008 to 2010 as a population base for estimates	Overall, 18% of cancer survivors reported financial hardship and 37% of the uninsured reported financial hardship. Of those, 39% (95% CI = 34–45%) would be Medicaid eligible under the Affordable Care Act. It will be important to monitor the effects of the Affordable Care Act on insurance coverage, access to care and out-of-pocket burdens for cancer survivors as the Affordable Care Act continues to be implemented.
Dieluweit, 2011 Level III—B	German Childhood Cancer Registry (GCCR) University Ulm Age at diagnosis 15–18 years Mean age 15.8 (0.9) Age at study: 20–46 years Mean years since diagnosis 13.7 (6.0)	Cross-sectional Aim: To investigate the educational and professional achievements of German long-term survivors of adolescent cancer	Cancer sites: Leukemia Lymphoma CNS tumors Neuroblastoma Retinoblastoma Renal tumors Hepatic tumors Malignant bone tumors Soft tissue and other extraosseous sarcomas Germ cell tumors	The German Socio-Economic Panel (G-SOEP) study provided comparison data to the general population (age-matched controls) The German Childhood Cancer Registry was used to access medical records.	Survivors were significantly older at the commencement of their first employment (*M* = 21.8, SD = 3.6) than the G-SOEP participants (*M* = 19.9, SD = 2.4; *t*[1,167]=10.9, *p* < 0.001). A Cox proportional hazard model analysis also demonstrated significant differences between the survivors and the German Socio-Economic Panel sample for age at first employment; even after statistical control for high school graduations and achievement of college/university degrees, survivors were significantly older at their first employment compared to the age-matched sample [survivors vs. German Socio-Economic Panel] OR 1.90, 95% CI 1.67–2.17, *p* < 0.001).
Ekwueme, 2014 Level I—A	US national survey *N* = 1202 Age at interview 18–39 years 4.5% (3.3–6.0) Age range 18–80+ years Cancer survivors who were employed at any time since diagnosis.	Cross-sectional Purpose: To estimate lost productivity by assessing employment disability, health-related missed work days, and days spent in bed because of ill-health	Cancer site: Not given	The Medical Expenditure Panel Experiences with Cancer Survivorship Survey Did cancer interfere with: Daily activities outside work Ability to perform mental tasks Ability to perform physical tasks required by job Ability to perform mental tasks required by job Employment Change in work Ever felt less productive at work.	Results presented in aggregate: Many cancer survivors (*n* = 676; 58.3%) return to work and remain productive. However, for cancer survivors who were employed at any time since diagnosis, cancer and its treatment interfered with physical tasks (*n* = 168; 25.1%) and mental tasks (*n* = 103; 14.4%) required by the job, with 24.7% (169) of cancer survivors feeling less productive at work.
Guy, 2014 Level III—A	US national survey *N* = 1464 adolescent/young adult cancer survivors 86,864 adults without cancer Young adult cancer survivor age at last interview: 18–29: 11% 30–39: 21% Years since diagnosis: 0–9: 30.5% 10–19: 27.7% 20+: 41.9%	Cross-sectional Descriptive Objective: To use nationally representative data to estimate direct medical costs and indirect morbidity costs among adolescent and young adult cancer survivors, compared to people without a history of cancer.	Cancer sites: Not given	2008–2011 Medical Expenditure Panel Survey Data	Young adult cancer survivors had higher annual per person medical expenditures ($7417) than adults without a history of cancer ($4247). Annual excess lost productivity was estimated to be $2250 per young adult cancer survivor. Identifying ways to reduce disruptions in education and at work as young adult survivors transition out of treatment is important for reducing the excess burden of cancer.
Hamilton, 2013 Level III—B	Mount Sinai Medical Center New York & Hackensack University Medical Center New Jersey *N* = 181 men and women who had undergone hematopoietic stem cell transplantation (HSCT) 9–36 months prior to assessment Participants were at least aged 18 years (and older than 16 years) at the time of HSCT. 640 days post-transplant	Cross-sectional Objective: To examine whether the portion of survivors’ transplant paid by health insurance, which varies across individuals, and affects how much of the treatment costs they bear, would moderate the association between economic survivorship stress and health-related quality of life.	Cancer sites: Hematologic malignancies	Researchers created own 12 Question tool “Employment Stressors in Hematopoietic Stem Cell Transplantation (HSCT) Questionnaire” Persistent financial, employment, and insurance stressors.	Financial and employment difficulties need to be viewed as sources of chronic stress with implications for survivors’ health long after treatment has ended
Hammond, 2015 Level III—B	Canada *N* = 21 young adult: 13 women and 8 men 18–45 years of age Variable length of time since treatment	Qualitative Aim: To investigate the prevalence of three tricksterly themes expressed within young adults’ stories of cancer.	Cancer sites: Not given.	Performance of tricksterdom in cancer survivors’ narratives from a social constructionist perspective: Destabilizing social or cosmic order Challenging dominant expectations for human life Exploring alternate ways of viewing the world.	3 major themes: Uncertainty, subversion, and possibility.
Keegan, 2014 Level II—A	National Cancer Institute Surveillance, Epidemiology, and End Results Program Adolescent & Young Adult Health Outcomes and Patient Experience study *N* = 464 adolescent/young adults survivors Age at diagnosis: 15–19 years: 62 (13.3%) 20–29 years: 195 (41.9%) 30–39 years: 208 (44.7%) 15–35 months after diagnosis.	Descriptive Aims: To determine young adult cancer survivors and non-cancer-related medical care in a 12-month period, and to examine sociodemographic and cancer-related factors associated with medical care use among survivors 15–35 months after diagnosis.	Cancer sites: Germ cell; acute lymphoblastic leukemia; non-Hodgkin lymphoma; sarcoma	Source of health insurance General Health Date of last treatment Quality of care Financial support Need for information	Employment categories: Unemployed 71—15.3% PT working/studying 92—19.8% FT working/studying 266—57.2% Other/unknown 36—7.7% Adolescent/young adult cancer survivors with current health insurance were nearly five times more likely to receive cancer-related care than those without health insurance (OR = 4.9; 95% CI = 1.7–13.8)/
Kim, 2013 Level III—C	Online forum 164 cancer-related blogs from *Planet Cancer* by 46 young adult cancer survivors. Age of participants at time of diagnosis or research was not given. *N* = 34 females—136 blogs; 12 males—28 blogs Length of time since diagnosis not given.	Qualitative Purpose: To explore the experiences and ganin a better understanding of young adult cancer survivors by examining their blogs.	Cancer sites not given	Survivors blogged about career and employment issues	Themes: life being affected by physical burdens, future prospects, and uncertainty, creating a positive attitude, and the paradoxical nature of cancer survivorship. Blogs provide support when survivors are isolated or physically unable to interact.
Kirchhoff, 2012 Level III—B	Behavioral Risk Factor Surveillance System (BRFSS) dataset USA, the District of Columbia, Puerto Rico, Guam, and Virgin Islands *N* = 1198 young adult cancer survivors between ages 20–39 years of age: 218 (age 20–29 years) 980 (age 30–39) Mean time since dx 7.4 years (SD 3.8)	Cross-sectional Aim: To determine how marital status is affected for young cancer survivors diagnosed ages 18–37.	Cancer sites: Not given	The Behavioral Risk Factor Surveillance System is an annual, nationally representative random-digit telephone survey of non-institutionalized adults ages 18 or older in the USA, DC, Puerto Rico, Guam, and Virgin Islands. The CDC-HRQOL-4 “Healthy Days Measure” was also used	Young adult cancer survivors were older than controls [33.0 (SD = 3.8) vs. 30.0 (SD = 4.0); *p* < 0.001). Survivors were employed less often (61 vs. 67.4%; overall *p* < 0.001). Survivors reported being 77% more likely to be divorced or separated among those who had every been married (survivors 18% vs. controls 10%; RR 1.77, 95% CI 1.43–2.19, *p* < 0.001) than controls.
Lewis, 2012 Level III—B	MD Anderson Cancer Center and Sisters Network *N* = 33 African-American breast cancer survivors Mean age at diagnosis: 37.39 Age range 25–45 years of age. Variable duration post-diagnosis Participants were at least 1 year post-diagnosis, off active treatment other than hormonal therapy.	Qualitative Objective: To explore the impact of cancer on women’s living situations, employment, relationships, fertility, and sexuality.	Cancer site: Breast	Semi-structured phone interviews 45–60 min 141 items focusing on impact of cancer on living situations, employment, relationships, fertility, and sexuality.	26% believed treatment interfered with employment Change in job due to cancer: Lost job 6% Mild/moderate negative impact on job 18% Positive impact/supportive workplace 24% No change 46% Not working outside home 6%
Love, 2012 Level III—B	Online forum Open to any young adult affected by cancer across the treatment spectrum. (U of Texas, Austin) *N* = 350 randomly sampled posts from 2007 to 2010 Unknown duration post-diagnosis	Qualitative Research question: What are the types of messages related to psychosocial needs being shared within the community?	Cancer sites: Not given	Speech events or types of talk: Exchanging support Coping Describing experiences Enacting identity Communicating membership	After treatment ends, survivors reported struggling with depression, strained relationships, and maladjustment to work, although some described a more meaningful outlook. Promotion of online support by care providers could provide additional support to individuals in need.
McCorkle, 2006 Level III—A	Southern New England tumor board *N* = 208 Median age at dx 39 years (range 29–92 years); Median age at time of survey 54 years (range 29–92 years) The average length of survival post initial diagnosis was 13.9 years (median = 13).	Cross-sectional Population-based survey Purpose: To describe the prevalence and correlates of depressive symptoms among women who have survived cervical cancer for 5–25 years.	Cancer site: Cervical 70% employed full time or functioning as homemakers	The Center for Epidemiological Studies-Depression Scale (CES-D)	Pain and post-radiation diarrhea predispose a sub-group of cervical cancer survivors to lingering problems that interfere with their ability to work. Researchers reported that this finding highlights the importance of adequate and appropriate management of cancer treatment-related symptoms during the extended or permanent survival stage.
Parsons, 2008 Level III - B	Toronto, Canada *N* = 14 bone tumor survivors (8 men; 6 women) Age at diagnosis: 16–35 years 5 years after diagnosis	Qualitative Objectives: To characterize the lived experiences of illness of people with osteosarcoma; To characterize the lived experiences of resuming vocational pursuits in the context of osteosarcoma; To understand and explain the relationship between these experiences.	Cancer site: Bone	In-depth interviews Topic areas: Vocational experiences and plans pre-diagnosis and post-treatment; Daily routines ‘then’ and ‘now’ Arriving at diagnosis Illness experiences	Respondents recounted being engaged in three kinds of work: illness work, identity work, and vocational work. All three types of work were intricately interwoven with illness work occurring during active cancer treatments, which was described as a transformative experience. Participants felt changed from who they were prior to cancer and when they returned to their respective vocations, they reported a changed relationship to work. Transformation of identity repositioned survivors differently socially, psychologically, and physically.
Parsons, 2012 Level III—A	National Cancer Institute’s Adolescent and Young Adult Health Outcomes and Patient Experience Study *N* = 463 Age at diagnosis (years): 15–19: 16 (31%) 20–24: 17 (25%) 25–29: 28 (29%) 30–34: 24 (27%) 35–39 21 (26%) Within 3 years of diagnosis	Observational cohort Purpose: To examine the impact of cancer on work and education in a sample of adolescent and young adult patients with cancer.	Cancer sites: Germ cell Non-Hodgkin’s lymphoma Hodgkin’s lymphoma Acute lymphocytic leukemia Sarcoma	Survey: What is your current school or employment status? Indicate what kind of impact your cancer has had on plans for education and plans for work.	Greater than 50% of survivors who were working or in school full time before diagnosis reported a problem with work/school both at 6–14 and at 15–35 months after diagnosis. In the follow-up survey, 30% of survivors working full time before diagnosis reported problems with “paying attention” at work/school. Further, at 15–35 months after diagnosis, 53% (*N* = 205) of all survivors reported problems with “forgetting”, while 28% (*n* = 107) reported troubles “keeping up with work or studies.”
Rabin, 2013 Level III—B	Hospital-based tumor registry in Rhode Island *N* = 20 young adult cancer survivors between 18 and 39 years of age. Mean age 33.5 years. Diagnosed within 10 years	Qualitative Objective: To develop an in-depth understanding of the preferred content and format of psychosocial and behavioral programs for those diagnosed with cancer during young adulthood.	Cancer sites: Breast (10%) Melanoma (10%) Thyroid (45%) 85% employed.	In-depth, semi-structured individual interviews Topic areas: Types of programs that might be helpful Delivery of programs Barriers to participation	>50% of participants advocated for using an online forum, chat room, or social networking site to receive support from other young survivors and behavioral counselors.
Rozmovits, 2014 Level III—B	UK *N* = 20 men and 19 women 28–68 years at diagnosis 33–87 years at the time of interview 5–9 years after diagnosis *[For this review, results from the young adult cancer survivor participants, aged 15–39 at time of diagnosis, were exclusively used.]*	Qualitative Aim: To consider aspects of the distress expressed by colorectal cancer patients in their personal narratives of illness and to produce a more detailed account of the illness’s impact on their identities and self-understanding.	Cancer site: Colorectal	Sample was referred for In-depth narrative interviews by Database of Individual Patients’ Experiences (DIPEx), an Internet resources based on illness narrative interviews	Major theme: loss of adulthood Sub-themes: loss of professional identity; loss of ability to socialize; loss of dignity, privacy, and independence. Management of bowel symptoms interferes with job performance and social expectations about professional behavior.
Rutskji, 2010 Level II—A	Cancer Registry of Norway: Testicular cancer survivor (TCS) Controls: Gallup Institute of Norway *N* = 1326 Mean age at survey 44.7 years (range 23–75 years); Mean age at orchidectomy 33.3 years (range 15–64 years) 11.4 years mean time after diagnosis (range 4.3–21.4 years)	Cross-sectional Objective: To explore approach and avoiding coping strategies in long-term testicular cancer survivors.	Cancer site: Testes Work status = paid work & self-employed vs. unemployed or pensioned	Research Questions: What is the pattern of approach-avoidant coping in testicular cancer survivors, and what are the differences in somatic and mental morbidity between testicular cancer survivors with more avoidance vs. more approach coping? What variables are significantly associated with approach/avoidance coping? Do testicular cancer survivors use more approach coping than a normative sample of men from the general population?	Approach and avoidance are 2 major coping strategies. Approach coping implies facing the stressor and making active efforts to manage it. In contrast, avoidance coping is characterized by passive, suppressive, and disengaged attitudes toward the stress. Paid work, self-employed testicular cancer survivors used more approach coping styles *N* = 966 (88)
Wettergren, 2003 Level III—A	Stockholm County Council’s database *N* = 121 Hodgkin’s lymphoma survivors Mean age at diagnosis 33 years Mean age at time of study 47 (11.9). Median time from Hodgkin’s lymphoma diagnosis to interview was 14 years (range 6–26 years).	Cross-sectional Descriptive Comparative Aim: To use an individual approach in evaluating quality of life in long-term survivors of Hodgkin’s lymphoma and their view of what impact the disease has had on life.	Cancer site: Hodgkin’s lymphoma	Extended version of the Schedule for the Evaluation of the Individual Quality of Life-Direct Weighting (SEIQoL-DW)	The most important areas in life, nominated by more than 50% of the Hodgkin’s lymphoma survivors as well as the control group, were family, personal health, work and relations to other people.
Yabroff, 2012 Level I—A	US national survey *N* = 884 Age category 18–44 (23.7%) Most cancer survivors were diagnosed 6 or more years prior to survey (52.7% for 18–64 years; 59.7% for 65+ years)	Cross-sectional Aim: To evaluate the association between cancer survivorship and service use frequencies and patient time costs.	Cancer sites: Not specifically given; included all.	The Medical Expenditure Panel Survey with Cancer Survivorship Supplement	Cancer care was typically more aggressive in younger than older cancer patients, potentially resulting in greater medical cost, productivity loss, late and long-term effects. Working young adults experience different types of late effects than survivors diagnosed with cancer at older ages.
Yanez, 2013 Level III—B	Tuluna, an online Research Panel *N* = 335 Mean age = 31.8 Stratified by cohort/time post-active treatment (months): 0–12 (118) 13–24 (98) 25–60 (106) Within 5 years of diagnosis	Cross-sectional Purpose: To explore whether age and cancer-related education/work interruption interacted with distress.	Cancer Sites: Breast Cervical Melanoma Lung Colorectal Thyroid Testicular	Impact of Event Scale (IES) Patient-Reported Outcomes Measurement Information System (PROMIS) CR work interruption was assessed by a single author-constructed item: ‘did you stop working because of your cancer?’ Yes/no	67.1% of the sample did not stop working because of cancer. Highest level of distress -13–24 months attend to cancer-related distress beyond the completion of treatment; target interventions such as psycho-social services.

^a^Dearholt, SL, & Dang, D. Johns Hopkins Nursing Evidence-Based Practice: Models and Guidelines, 2nd Ed, 2012.

## Results

The 23 eligible publications included young adult cancer survivor populations from the USA, Norway, Sweden, Germany, Canada, Switzerland, The UK, and Europe. Seven publications were cancer site-specific: osteosarcomas, colorectal cancer, testicular cancer, Hodgkin’s lymphoma, breast cancer, and cervical cancer [14, 15, 19, 20, 24, 25, 32]. Two of these studies emphasized hematopoietic stem cell transplants [22, 27]. Five articles [16, 21–23, 29] described survivors during the first 5 years of survivorship while nine publications [11, 14, 15, 17, 19, 25–27, 32] examined survivorship in the long-term, greater than 5 years after initial diagnosis and treatment. The remainder of articles covered the complete span of young adult cancer survivorship, both short and long-term. Most publications utilized quantitative research methods. Seven [8, 20, 24, 28, 30–32] articles featured qualitative designs. The final group of articles meeting eligibility criteria were published between 2003 and 2015. The search identified two large cohort studies that led to more than one publication: The Medical Expenditure Panel Survey (MEPS) [11–13, 18] and the Adolescent and Young Adult Health Outcomes and Patient Experience (AYA, HOPE) Study [16, 21]. Publications meeting eligibility criteria from these large databases are included in this review. Each publication was thoroughly reviewed to determine if the study’s sample included young adult cancer survivors diagnosed between the ages of 15 and 39, and the length of time since cancer diagnosis. Additionally, inclusion of employment, or work, with related findings was abstracted.

This review revealed that young adult cancer survivors ultimately return to work (Table 1). However, it may not be the same work if physical or cognitive changes occurred because of cancer treatments. Work from a psychological perspective is viewed as mental exertion that is difficult, exhausting, or entails creative effort [32]. Only publications conceptualizing work as a synonym for paid employment were considered. Articles describing factors that influence work or employment were included in this analysis to provide a comprehensive appraisal of the work-related issues for young adult cancer survivors. Distress often results from economic challenges presented by costs associated with cancer care along with ability to work and earn wages. Health insurance provided by employers remains an important decision as young adult cancer survivors seek to obtain employment or return to work after treatments to ensure lifetime access to healthcare. The distress of financial burdens can also affect intimate relationships and overall quality of life.

**Fig. 2:**
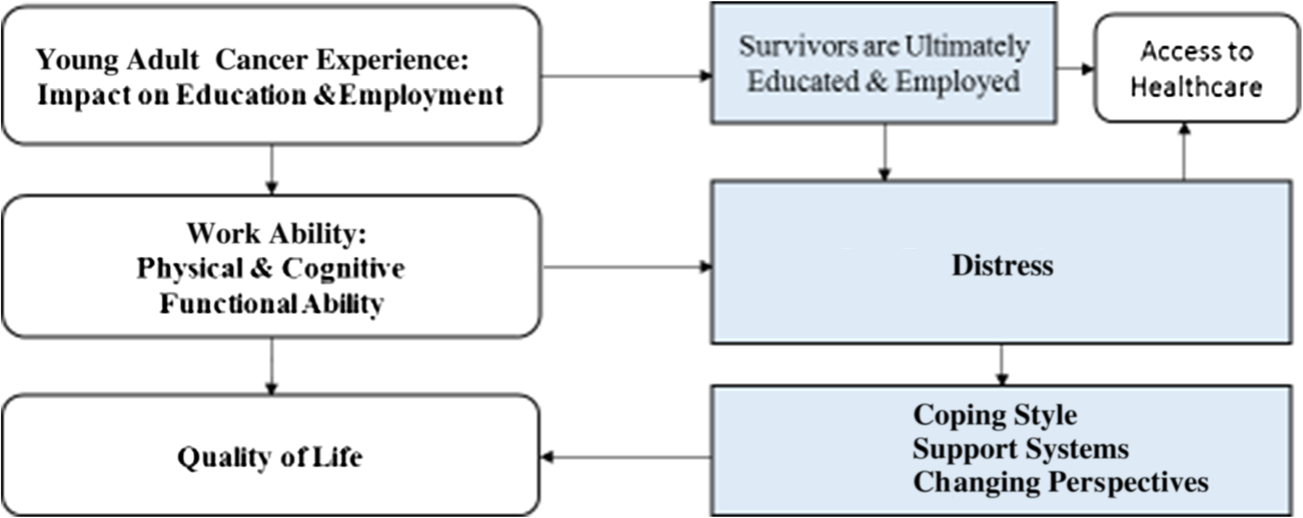
Relationships among themes

Two primary themes emerged from this review, work ability and distress. Subcategories within each theme provided a clearer understanding about the influences and impact of physical and cognitive functional abilities from cancer or its treatments on work ability. Coping style, support systems, and changing perspectives about work and life underpinned survivors’ actions and reactions to cancer-associated distress (Fig. 2).

### Work ability

Dieluweit and investigators [17] surveyed 820 survivors during adolescence (mean age at diagnosis 15.8 years of age). Survivors were found to be significantly older at commencement of their first employment (*M* = 21.8, SD = 3.6) in comparison to the *German Socio-Economic Panel Study* participants (*M* = 19.9, SD = 2.4; *t*[1,167] = 10.9, *p* < 0.001). However, ultimately survivors were as likely to graduate from university or to be employed as controls without a history of cancer.

*The Medical Expenditure Panel Survey with Cancer Survivorship Supplement* by Yabroff and colleagues [11] is a nationally representative survey detailing the burden of cancer, including access to healthcare, employment patterns in survivors, lost productivity, financial issues and the psychosocial impact on survivors and their families. The supplement indicated that cancer care is typically more aggressive in younger than older cancer patients, potentially resulting in greater medical cost, productivity loss, late and long-term effects. Working young adults with a cancer history may spend more years living with lasting effects of cancer or its treatment and experience different types of late effects than survivors diagnosed with cancer at older ages. Similarly, Guy et al. [18] also studied *The Medical Expenditure Panel Survey* to understand the economic impact of cancer on young adult cancer survivors. The researchers found that surviving cancer during young adulthood is associated with substantial economic burden. Young adult cancer survivors had an excess annual medical expenditure of $3170/person and excess annual productivity losses of $2250/person. The conclusion from this investigation stressed the need to ensure access to lifelong risk-based follow-up care.

The Centers for Disease Control and Prevention agrees with the above findings in their report *Medical Costs and Productivity Losses of Cancer Survivors—United States, 2008–2011* by Ekweme et al. [12]. Cancer survivors in this study were stratified into two large age groups, 18 to 64 years and 65+ years. The researchers noted that many cancer survivors (*n* = 676; 58.3%) return to work and remain productive. However, for cancer survivors who were employed at any time since diagnosis, cancer and its treatment interfered with physical tasks (*n* = 168; 25.1%) and mental tasks (*n* = 103; 14.4%) required by the job, with 24.7% (169) of cancer survivors feeling less productive at work. Other investigations support this statement as shown in the work ability subcategories: physical and cognitive functional ability.

#### Physical functional ability

McCorkle and colleagues [19] used a quality of life framework to conduct a population-based survey of women identified via a state tumor registry in southern New England to describe the prevalence and correlates of depressive symptoms among women who survived cervical cancer from 5 to 25 years (*N* = 25). Self-reported impact of cervical cancer on ability to work was included in the survey tool. Median age at diagnosis was 39 years; median age at time of survey was 54 years. Difficulty in ability to work increased the odds of depressive symptoms (4.46, 95%CI 1.44–13.76). Results indicated that pain and post-radiation diarrhea predispose a subgroup of cervical cancer survivors to lingering problems that interfere with their ability to work. Researchers reported that this finding highlights the importance of adequate and appropriate management of cancer treatment-related symptoms during the extended or permanent survival stage.

Rozmovits and Ziebland [20] used a qualitative approach to explore relationships between *The Civilizing Process* by theorist and author Elias [33] with the experiences of colorectal cancer survivors in the UK. A sense of adulthood in relation to employability and professionalism was part of this alignment between Elias’s work and survivors’ narratives. The researchers noted that according to Elias, for individuals to be considered civilized, they need to exercise control over bodily impulses. Twenty men and 19 women were interviewed who were initially diagnosed at ages ranging from 28 to 68 (33–87 years at time of interview). An overarching theme of the loss of adulthood emerged. Subthemes included loss of professional identity, loss of ability to socialize, and loss of dignity, privacy, and independence. Control over bowel habits emerged as an important issue for all participants, with or without a stoma. The researchers found that urgent response to sudden bowel evacuation in work-related situations takes a toll on job performance as well as fundamental aspects of adult identity linked to social expectations about professional behavior. The researchers also noted that younger people who had to abandon their careers suddenly were challenged in seeking to fill that void.

#### Cognitive functional ability

Utilizing data from the National Cancer Institute’s Adolescent and Young Adult Health Outcomes and Patient Experience (AYA HOPE) Study, Parsons and colleagues [21] examined factors associated with a return to full-time employment or school after cancer diagnosis with a belief that cancer had a negative impact on an individual’s work or educational plans. All study participants were between the ages of 15 and 39 at time of cancer diagnosis. The analysis focused on full-time workers/students at time of diagnosis. Of the 463 patients in the AYA HOPE study who completed initial and follow-up surveys, more than 72% of patients who reported working or being in school full time before diagnosis had returned to full-time work 15–35 months’ post-diagnosis. More than 50% of all patients working or in school full time before diagnosis described problems with cognition at 6–14 months after diagnosis and at 15–35 months after diagnosis. For example, in the follow-up survey, 30% of patients working full time before diagnosis recounted difficulty “paying attention” at work/school. Further, 15–35 months after diagnosis, 53% (*N* = 205) of all patients reported “forgetting,” while 28% (*n* = 107) had difficulty “keeping up with work or studies”. Similarly, Prasad et al. [34] found in the Childhood Cancer Survivor Study database cognitive and behavioral functional problems in long-term survivors (5+ years since diagnosis) diagnosed during adolescence or early adulthood (*N* = 1334 survivors 15–21 years of age at diagnosis). Self-reported difficulties with task efficiency increased risk for unemployment (OR, 2.93; 95%CI, 2.28 to 3.77), compared to survivors without problems.

### Distress

Hamilton and colleagues [22] mailed questionnaires and conducted telephone interviews to determine associations between economic stress and health-related quality of life in 181 survivors *(M* = 640 days’ post-transplant). Seventy-three percent of participants (older than 16 years at time of hematopoietic stem cell transplantation) were working at the time of their diagnosis or transplant; however, only 44% were employed during the study. Among the employed survivors, the most common financial problem was a pay cut or lost income due to illness (reported by 67% of participants; 46% found this to be very or extremely upsetting). The next most common problems included going on disability (63%) and needing to take a paid leave of absence (55%). All but one participant had health insurance at the time of transplant. On average, insurance stress experienced during the course of illness or transplant was low as indicated by eight items measured on a five-point scale *(M* = 3.43, SD = 4.68). The results from this study suggested financial and employment difficulties need to be viewed as sources of chronic stress with implications for survivors’ health long after treatment has ended.

Aksnes, Hall, Jebsen, Fossa, and Dahl [14] examined fatigue, mental distress, and quality of life in extremity bone tumor (EBT) survivors at long-term follow-up compared to gender and age-matched control participants with a history of Hodgkin’s disease or testicular cancer in Norway. Normative data was also used for comparison in this study. Mean age at diagnosis ranged from 16 to 25 among the survivors studied. Findings revealed the EBT survivors did not differ from the other survivor groups as to the level of education and employment status. In the multivariate analyses of the combined survivor and normative data, a low level of education and not being employed were associated with mental distress. Neither age at diagnosis nor time since diagnosis was associated with distress among the survivors.

#### Coping

Yanez, Garcia, Victorson, and Salsman [23] explored interaction of cancer-related distress with age and interruption of education or work in young adult cancer survivors (mean age = 31.8). The Impact of Event Scale (IES) was used to determine work interruption and interaction between cohort (time post-active treatment 0–12 months; 13–24 months; 25–60 months) and cancer-related education/work interruption on distress. IES is a 15-item self-report measure of intrusive and avoidant cognitions frequently used in evaluating stress reactions after traumatic experiences. Intrusive cognitions can be ordinary autobiographical memories, or spontaneous flashbacks, whereas, avoidant cognition is a defense mechanism used to avoid coping. Cancer-related work interruption was also assessed by a single author-constructed item: “Did you stop working because of your cancer?” 67.1% of the sample did not stop working because of cancer. Survivors in the 13–24 and 25–60-month cohorts reporting education/work interruption were significantly more distressed than those not reporting education/work interruption (*p* < 0.05). After adjusting for physical symptom level and gender, a three-way ANCOVA revealed significant effects: *F*(2,310) = 9.49, *p* < 0.05, global impact *F*(4, 310) = 9.95, *p* < 0.001, of cancer-related education/work interruption *F*(1,310) = 4.03, *p* < 0.05 on distress. Attending to cancer-related distress beyond the completion of treatment was recommended in the conclusions. Target interventions such as psychosocial services were suggested.

Rutskij et al [15] conducted a cross-sectional follow-up study of unilateral orchietomized testicular cancer survivors in Norway using the Brief Approach/Avoidance Coping Questionnaire (BACQ) among others. The participants were an average age of 33.3 years at time of diagnosis; mean age at time of survey was 44.7 years. Despite being considered cured of testicular cancer, this sample of survivors continued to demonstrate increased levels of anxiety compared to healthy male controls. Approach and avoidance were the two major coping strategies studied during this investigation. Approach coping implies confronting stressors and making active efforts at management. Alternatively, avoidance coping is characterized by passive, suppressive, and disengaged attitudes toward stressors. The researchers found that survivors who were employed had higher levels of approach coping style, considered a healthier response to stressful situations.

#### Support

Lewis and colleagues [24] explored psychosocial concerns in semi-structured telephone interviews with 33 African-American breast cancer survivors (mean age at diagnosis 37.39). Almost all women (*n* = 31) worked outside the home at diagnosis with nearly half of the participants (46%) reporting that cancer had no impact on their careers. Twenty-four percent of the participants indicated that employment in a supportive workplace had a positive impact on their careers. Another 18% of women believed, however that cancer had a mild/moderate negative impact on their careers, including two women (6%) who reported job loss due to cancer. The remaining 6% of women were not working outside the home.

In Sweden, Wettergren et al [25] evaluated individual quality of life in long-term survivors (median time from diagnosis to interview was 14 years; mean age at time of study = 47 (11.9) of Hodgkin’s lymphoma (HL) and their views about disease impact. Findings were compared with a randomly selected control group from the Stockholm County Council Database. No significant difference was found between the HL survivors’ (5.4, SD 0.9) and controls’ (5.3, SD 0.7) quality of life index scores ranging on a scale from one to seven. The most important areas in life, identified by more than 50% of the participants in both groups, were family, personal health, work and relations to other people. The investigators concluded that special attention should be given to survivors’ relationships with family and close friends, work-related issues, and late effects from treatment.

Distress may also impact marital relationships. Marriage and divorce among young adult cancer survivors (*N* = 1198) was investigated by Kirchhoff et al [26] with comparison to young adult controls without a cancer history. The 2009 Behavioral Risk Factor Surveillance System (BRFSS) dataset was studied to determine how marital status affects young adult cancer survivors diagnosed between ages 18 and 37 (mean 33.0 (3.8)) with an average time since diagnosis at 7.4 years. Sixty-one percent (731) of survivors were employed (full or part-time status was not given); 19.7% (213) choose not to be in the labor force; 11.1% (125) were out of work or unemployed; 8.2% (127) were unable to work. The investigators found that even with adjustment for education in the regression models, young adult survivors were more likely to divorce or separate than the controls. Financial stressors may contribute to divorce or separation for young adult cancer survivors. The investigators were unable to determine how financial status preceding or during cancer treatment affected martial outcomes. They concluded that many young adult survivors confront financial hardship due to missed work and/or lost income that negatively affect marriage.

Keegan et al. [16] studied barriers to access of medical care in 465 adolescent and young adult cancer survivors identified through the population-based SEER program cancer registries. Eligible participants were 15–39-year-old residents of eight geographic regions (Detroit; Seattle; Los Angeles; San Francisco; Sacramento; Orange County, CA, Iowa; Louisiana) who were newly diagnosed during 2007–2008. Young adult cancer survivors without insurance were generally less likely to see all types of doctors, particularly oncologists. Among young adult survivors who did not report a doctor’s visit in the past 12 months, the three most common reasons for no care were high cost/no insurance (44%), they felt they did not need follow-up care (40%) or their physician said they did not need follow-up care (28%).

Davidoff, Hill, Bernard, and Yabroff [13] examined potential improvements in access to insurance for cancer survivors through adult Medicaid expansions and premium tax credits in the new insurance marketplaces under the Affordable Care Act (ACA). The Medical Expenditure Panel Survey Household Component (2008–2010) was used to sample 2527 cancer survivors; 24.4% of the sample was in the young adult age category 18–44. Overall, 18% of cancer survivors reported financial hardship and 37% of the uninsured reported financial destitution. The experience of cancer survivors prior to the ACA indicates that many faced substantial out-of-pocket burdens even with insurance. Given the heavy personal financial burden and access barriers faced by cancer survivors, it is expected that many of those without current employment-related insurance would participate in Medicaid or the marketplaces. Plans purchased through the marketplace are required to provide adequate networks but may limit the number of providers to maintain affordability, which may impede cancer survivors’ access to oncology specialists. The researchers note the importance of monitoring the changing landscape of insurance coverage, access to care, and uncovered medical expenses for cancer survivors as healthcare access continues to be revamped in the USA.

#### Changing perspectives

Bieri and colleagues [27] studied young adult cancer survivors after allogeneic hematopoietic SCT (*N* = 124) in Switzerland using the *European Organization for Research and Treatment of Cancer Quality of Life Questionnaire-C30* and *the Functional Assessment of Cancer Therapy-Bone Marrow Transplant* tools. Survivors’ median age at diagnosis was 34, with median time since treatment of 7 years. A control group of healthy participants was not recruited or sampled for comparison in this study. The researchers found that age and employment status were significantly associated with global quality of life. Among survivors employed full time, 73% reported good quality of life as opposed to 28% working part time, and 22% of those on disability insurance (HR 0.35 (95%CI 0.22–0.58) *p* < 0.0001). Younger than 25 years of age at hematopoietic stem cell transplantation and return to full-time employment were the only parameters in this study that were significantly associated with a better perception of health-related quality of life in comparison to other patient characteristics, such as age or gender.

Using qualitative methods, Parsons et al. [32] studied young adult primary bone cancer survivors in Toronto, Canada. The eligible participants were diagnosed at ages 22, 25, and 30 and interviewed at 27, 31, and 35, respectively. The researchers prompted participants to reflect about returning to work. Respondents recounted being engaged in three kinds of work: illness work, identity work, and vocational work. All three types of work were intricately interwoven with illness work occurring during active cancer treatments, which was described as a transformative experience. Participants felt changed from who they were prior to cancer and when they returned to their respective vocations, they reported a changed relationship to work with a different sense of themselves from when they had left the workplace. Transformation of identity repositioned survivors differently socially, psychologically, and physically. The researchers recommended clinicians adopt a sophisticated approach when discussing plans for returning to work with survivors. Improvement of programs within the workplace that are tailored to meet individual needs were also encouraged.

Hammond, Reese, and Teucher [28] used a qualitative approach to produce an accurate accounting of relationships between personal stories of cancer and cultural understanding of illness. Twenty-one young adults were individually interviewed using a semi-structured guide. Uncertainty and possibility were two themes that emerged from this study. Participants expressed uncertainty in relationship to cancer diagnosis, treatment, and prognosis, but also maintained uncertainty when discussing the future beyond treatment. However, possibility was the theme linked most strongly with work among survivors whose career perspectives and life priorities had changed due to cancer experiences. Alternative career or employment opportunities for future endeavors fit well into the possibility theme.

Bellizzi et al. [29] studied the Adolescent and Young Adult Health Outcomes and Patient Experience (AYA HOPE) data to identify the negative and positive impact of cancer on adolescent and young adult cancer survivors in three age categories: 15–20 years (33.8%), 21–29 (39.9%), and 30–39-year-olds (38.2%). The most prevalent negative life domains young adults with cancer reported were specific to future plans (financial situation, plans for having children, plans for working) as well as body appearance and sense of control over life. Regarding future plans, all three age categories reported that cancer had similar level (∼46% of the sample) of positive impact for the future and goal setting.

### Generating supportive relationships

Psychosocial concerns were explored in semi-structured telephone interviews with 33 African-American breast cancer survivors (mean age at diagnosis 37.39) by Lewis and colleagues [24] using a 49-question tool containing demographic information including profession and working status outside the home. Several questions inquired about support received during treatments with reflection about support that was missing. Impact of the cancer experience on sexuality and fertility was also included. The majority (64%) of participants were self-identified as being in a white collar occupational category. Twenty-four percent of women reported that cancer had a positive impact on their work life and that co-workers were supportive during cancer treatment; however, one third wished for more emotional support from family or intimate partners, and that need did not diminish over time. The researchers had partnered with a national advocacy organization *Sisters Network Inc.* to create a peer counseling program *SPIRIT* (Sisters Peer Counseling in Reproductive Issues after Treatment) and concluded that psychosocial interventions from such an organization, or a patient navigator model, may provide support to young African-American breast cancer survivors. Mental healthcare or counseling seemed to be especially needed by this young survivor group, which could potentially be provided at the worksite, but was not suggested by the investigators.

Rabin, Simpson, Morrow, and Pinto [30] sought to obtain an in-depth understanding of the preferred content and format of psychosocial and behavioral programs for those diagnosed with cancer during young adulthood. The researchers conducted semi-structured individual interviews with 20 young adult cancer survivors (5 men; 15 women) between 18 and 39 years of age (mean = 33.5 years). Eighty-five percent of the participants were employed during the study. About half of the survivors reported that interventions delivered via the Internet have the potential to maximize convenience. Over half (percentage not given) of the participants advocated for using an online forum, chat room, or social networking site to communicate with, and receive support from, other young survivors and behavioral counselors. Most participants felt that a behavior change intervention delivered via telephone would provide an enhanced degree of support and social connection.

Love et al. [31] studied 350 randomly selected messages, or speech events, related to the psychosocial needs of young adult cancer survivors in an online environment hosted by the University of Texas, Austin. The forum is open to any young adult affected by cancer across the treatment spectrum. It was expressed by members that once treatment ends, survivors struggle with depression, strained relationships, and maladjustment to work, although others describe a more meaningful outlook. The researchers concluded that promotion of online support through care providers could attract more individuals in need of assistance or counseling.

In a qualitative study by Kim and colleagues [8], 164 blogs submitted to the *Planet Cancer* website by 46 young adult cancer survivors were examined. Several major themes were reported with connections between cancer survivorship and work, for example: life being affected by physical burdens, prospects and uncertainty, creating a positive attitude, and the paradoxical nature of cancer survivorship. Loss of control experienced by the young adult cancer survivors studied was found to be related to external factors such as career, education, and family planning. Internet-based cancer support services, specific to young adult cancer survivors, were viewed as being a familiar mode of support with those who are of similar age and in similar situations.

## Discussion

By examining the effects of cancer and its treatments on work, this review fills an important gap in knowledge about young adults diagnosed with cancer between 15 and 39 years of age. This systematic review revealed that the cycle of functional ability impacting work productivity on employment is a strong source of distress affecting not only acquiring and maintaining a job but also financial security, access to healthcare, relationships, and quality of life. The work-related issues for young adult cancer survivors during a career trajectory are complex with physical and psychosocial implications. In the review provided, survivors initially experienced delays in obtaining education and employment due to cancer diagnosis and treatment. However, the related studies found that survivors become equal to healthy controls overall in achieving a successful career. Many young adult cancer survivors continue working throughout the treatment phase, although treatment schedules and side effects sometimes interfered with productivity. The reviewed literature also indicated that physical or cognitive consequences occasionally prompted a change in occupation. Alternately, while some survivors find work to be a return of normalcy, others describe a changed perspective that redefined future goals. The length of survivorship ranged from 1 year to 20 years or more, post cancer diagnosis or treatment in the literature reviewed. However, with limited research focusing on a very small component of the career development process, significant knowledge gaps related to the impact of cancer and associated treatments on career development still exist [35].

Considering comparability of cancer with non-cancer samples, as well as the effects of cancer on young adult survivors with older adult survivors from investigations not eligible for this review, we found contrasting results that are also optimistic. For example, in a Canadian study by Jeon [36], effects of cancer on work status and earnings in survivors aged 25–61 revealed smaller negative long-term effects on the work status of survivors diagnosed at 48 years of age and younger than the effect for the full cancer group. In an investigation comparing cancer survivors 28–54 years of age (*N* = 676) 2–6 years after diagnosis with non-cancer controls in Pennsylvania, Moran, Short, and Hollenbeak [37] found the extent of cancer’s long-term effects on employment unclear, a contradiction that could be explained by survivors who continue to work despite impairments and disabilities. Additionally, according to a meta-analysis by de Boer [38] and colleagues, cancer survivors in general are 1.37 times more likely to be unemployed than health control participants although age did not have a clear association with unemployment risk. These variations in results may be explained in a systematic review by Duijts et al [39], who found that cancer survivors require a period of adjustment to cope with work demands again upon return.

The limitations of the publications reviewed limit generalizability of findings for several reasons. Labor laws, national insurance programs, and benefits differ between the countries and states within the USA. In addition, several studies seemed to emphasize whether young adult cancer survivors with specific diagnoses were working rather than how they were functional and productive at work. The studies presenting changes in physical functioning were specific to types of cancer or treatments, and described consequences while neglecting to provide content about accommodations that will be needed for work. Since half of the eligible publications were at evidence level 3, experimental investigations are especially needed to strengthen the body of knowledge about young adult cancer survivors in the workplace. Five of the 23 publications [8, 12, 13, 28, 31] did not reveal the length time after cancer diagnosis or treatment, an important marker to determine long-term effects on work ability. However, strengths of the publications reviewed highlighted work as central to quality of life since it is pivotal to financial stability and quite often access to health insurance and ultimately continued healthcare. Young adult cancer survivors can now ponder the complexity of health insurance systems in the USA, as access to healthcare is important to consider during times of employment transitions.

## Conclusions

From this review, the resounding message for clinicians in the publications reviewed largely relate to the need for close monitoring of young adult cancer survivors to determine the presence of distress and/or depression. The aftermath of emotions following treatment is a particularly vulnerable time that may require additional time or services. It is also important to assess the interests of young adult cancer patients as well as their dreams and career aspirations. Inter-disciplinary collaboration is critical to understanding the process of returning to work in view of symptomology, and the potential need for accommodation (Table 2).

**Table 2:** Interventions and improvements for clinicians and occupational settings from reviewed publications

Clinicians	Occupational settings
Discuss plans for returning to work	Discuss plans for returning to work
Discuss alternate careers and employment opportunities if needed	Tailor work to accommodate individual needs
Refer for mental health counseling if needed	Provide Professional Employee Assistance services
Refer to secure online support forums, chat rooms, or social networking	Notify survivors of services available in the workplace

## Implications

To the best of our knowledge, no studies explored interactions between young adult cancer survivors and occupational and environmental health professionals in the workplace. Research is needed to investigate young adult cancer survivors regardless of cancer site to determine specific challenges encountered in the workplace over the course of a career, along with evidence-based strategies that contribute to success. Additionally, important to explore are support services available within the work arena, and if employed survivors are aware of the support available. Since technology was shown to be a flexible and convenient mode of communication for young adult cancer survivors to communicate, the use of online forums could be a feasible and effective method of providing support. Clearly, young adult cancer survivors are an integral part of the workforce. Determining physical and cognitive functioning and changes in needs over time are important directions for future research, formulation of workplace policies, and maintenance of a robust labor market.
